# Refractory immune thrombocytopenia treated with low-dose decitabine combined with recombinant human thrombopoietin or eltrombopag: Two case reports

**DOI:** 10.1097/MD.0000000000041449

**Published:** 2025-02-07

**Authors:** Yuxia Jiang, Yani Xu, Haiying Wu, Minxia He, Huifang Jiang, Xiaofeng Xu

**Affiliations:** a Department of Hematology, Tongde Hospital of Zhejiang Province, Hangzhou, P.R. China; b Department of Rehabilitation, Zhejiang Aged Care Hospital, Hangzhou, Zhejiang, P.R. China; c Department of Hematology, Hangzhou Red Cross Hospital, Hangzhou, Zhejiang, P.R. China.

**Keywords:** eltrombopag, immune thrombocytopenia, low-dose decitabine, refractory, thrombopoietin

## Abstract

**Rationale:**

Immune thrombocytopenia (ITP) is an autoimmune-mediated disorder caused by antibody-mediated platelet destruction and impaired platelet production by megakaryocytes. Treating refractory ITP remains a significant challenge.

**Patient Concerns and Diagnoses:**

We report 2 patients with refractory ITP.

**Interventions and Outcomes:**

Two patients received low-dose decitabine combined with recombinant human thrombopoietin or eltrombopag. Platelet counts in both patients increased to within the normal range.

**Lessons:**

Low-dose decitabine combined with recombinant human thrombopoietin or eltrombopag may exert a synergistic effect in the treatment of refractory ITP.

## 1. Introduction

Immune thrombocytopenia (ITP) is an autoimmune-mediated disease caused by antibody-mediated platelet destruction and impaired platelet production by megakaryocytes.^[1]^ Standard treatments include glucocorticoids, immunoglobulin (IVIG), immunosuppressive agents, thrombopoietin (TPO)-receptor agonists (RAs), splenectomy, and anti-D immunoglobulin.^[2,3]^ However, 20 to 30% of patients with ITP do not respond to any of these treatments.^[4]^ Managing refractory ITP remains a significant challenge for clinicians. The combination of low-dose decitabine (DAC) and recombinant human (rh) TPO or TPO-RA, which promotes platelet count elevation, has rarely been studied. Tang et al^[5]^ reported that the combination of low-dose DAC and rhTPO for refractory prolonged isolated thrombocytopenia following hematopoietic cell transplantation may more effectively improve the bone marrow microenvironment and platelet recovery than DAC alone. Here, we report 2 cases of refractory ITP resistant to both first – and second-line therapies. Notably, platelet counts returned to normal after treatment with low-dose DAC combined with rhTPO or eltrombopag.

## 2. Case 1

A 69-year-old male patient was admitted to our hospital on January 9, 2018, with thrombocytopenia persisting for >10 years. Over a decade earlier, his platelet count fluctuated between 50 × 10^9^/L and 60 × 10^9^/L, while white blood cell and hemoglobin counts remained within the normal range.

He had not sought medical attention for his condition. In June 2015, he experienced a cerebral hemorrhage with a platelet count of 8 × 10^9^/L and a blood pressure reading of 200/100 mm Hg. He underwent a craniotomy following a platelet transfusion and experienced good postoperative recovery. Based on his medical history and bone marrow examination results, he was diagnosed with ITP. He showed a temporary response to glucocorticoids. A combination of rhTPO (15,000 units/day, days 1–10) and IVIG (0.4 g/kg, days 1–5) significantly increased his platelet count to the normal range temporarily. However, without continued maintenance therapy, his platelet count declined to the range of 20–30 × 10^9^/L.

In January 2016, the patient received subcutaneous rhTPO injections (15,000 units/day for 7 days per month) combined with oral azathioprine (50 mg, twice daily), which stabilized his platelet count at approximately 50 × 10^9^/L.

**Figure 1.**
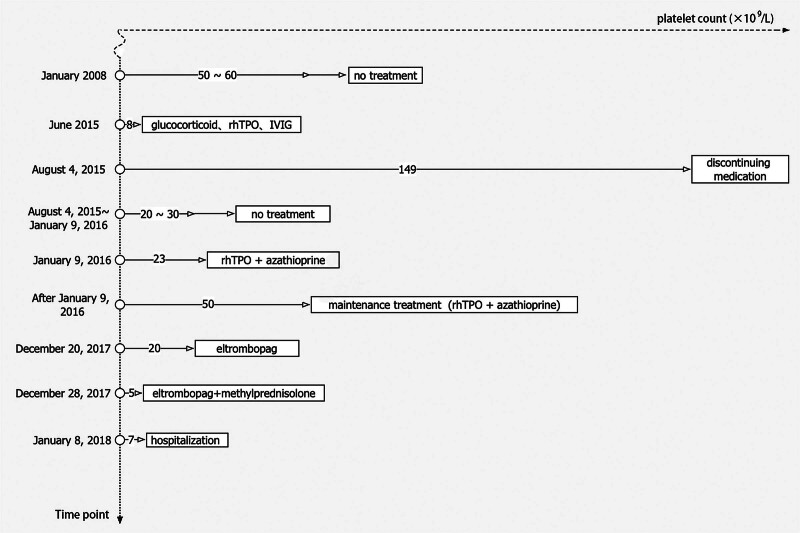
The patient’s medical history of treatment for ITP. ITP = immune thrombocytopenia.

On December 20, 2017, a routine blood test showed a platelet count of 20 × 10^9^/L, a hemoglobin level of 97 g/L, and a white blood cell count of 7.9 × 10^9^/L. Bone marrow puncture revealed a moderate number of nucleated cells, an increased number of megakaryocytes, and diminished platelet-producing function. Bone marrow biopsy indicated mild reticular fiber hyperplasia, slightly low hematopoietic tissue hyperplasia, and no evidence of abnormal hematopoiesis. Flow cytometry and gene expression analysis associated with myelodysplastic syndrome (MDS) did not reveal any abnormalities. He was admitted and treated with eltrombopag (50 mg per day). On December 28, 2017, he exhibited no platelet response, with a platelet count of 5 × 10^9^/L. Subsequently, he received eltrombopag (50 mg per day) combined with methylprednisolone (8 mg twice daily) for 12 days, resulting in a platelet count of 7 × 10^9^/L. He was later referred to our hospital for further treatment (Fig. 1). The patient did not take medications known to be associated with thrombocytopenia.

The patient had a 20-year history of hypertension, an 18-year history of gout, a 10-year history of diabetes mellitus, and an 18-year history of uremia with a 2-year history of peritoneal dialysis.

Physical examination revealed petechiae and ecchymoses on the skin without lymphadenopathy, hepatosplenomegaly, or lower sternum tenderness.

After admission, a routine blood test showed a white blood cell count of 7.7 × 10^9^/L, a lymphocyte level of 0.6 × 10^9^/L, a hemoglobin concentration of 80 g/L, a platelet count of 12 × 10^9^/L, and a reticulocyte percentage of 1.2%. Ferritin, folate, and vitamin B12 levels were all within the normal range. Biochemical examination revealed a urea nitrogen level of 32.78 mmol/L, creatinine at 561.9 µmol/L, and alanine aminotransferase at 12 U/L. Antinuclear antibodies and antineutrophil cytoplasmic antibodies were within normal ranges. However, the β_2_-microglobulin level was elevated at 18.50 mg/L (normal range: 1.01–3 mg/L), which was attributed to the patient’s renal failure. A B-mode ultrasound showed no abnormalities in the internal organs, including the liver, gallbladder, spleen, pancreas, retroperitoneal lymph nodes, and superficial lymph nodes. Despite receiving daily peritoneal dialysis (1.5% peritoneal dialysate exchanged 3 times daily with 2000 mL per exchange), his serum creatinine levels remained elevated, fluctuating between 500 and 600 µmol/L. Due to his hemorrhagic tendency and severe renal insufficiency, the patient was not a candidate for splenectomy.

**Figure 2.**
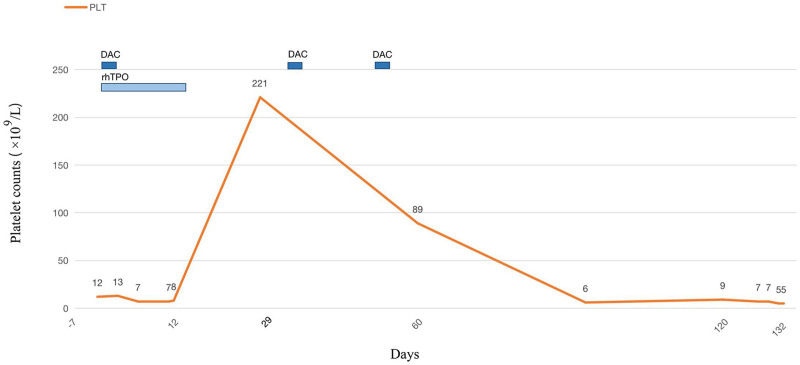
The first day of treatment is designated as day 1. The patient received low-dose DAC for 3 days and rhTPO for 14 days, resulting in a platelet count increaseto 221 × 10^9^/L by day 29. After 4 weeks, the patient was treated with the same-dose DAC without rhTPO, leading to a decline in platelet levels. DAC = low-dose decitabine, rh = recombinant human, TPO = thrombopoietin.

On January 17, 2018, the patient was treated with low-dose DAC (6 mg daily; 3.5 mg/m² per day, intravenous infusion) for 3 days and rhTPO (30,000 U, subcutaneous injection, every other day) for 14 days, with a cycle lasting 4 weeks. His platelet count increased to 221 × 10^9^/L by the end of the first cycle. The patient subsequently received 2 additional cycles with low-dose DAC alone (6 mg daily; 3.5 mg/m² per day for 3 days). However, by March 15, 2018, his platelet count began to decline, reaching 89 × 10^9^/L. No adverse events related to DAC, such as renal function deterioration, were observed. Due to financial constraints, the patient discontinued further diagnosis and treatment, leading to a gradual decrease in his platelet count to 5 × 10^9^/L (Fig. 2). Unfortunately, he eventually died from intracranial hemorrhage.

## 3. Case 2

A 50-year-old female patient was admitted to our hospital on June 23, 2022, with thrombocytopenia persisting for more than 1 year. One year earlier, she presented with abdominal pain and bloating, and a routine blood test revealed a platelet count of 31 × 10^9^/L, while white blood cell and hemoglobin levels were within normal limits. A bone marrow smear indicated an increased number of megakaryocytes, with 119 megakaryocytes identified across the entire slide, including 5 platelet-producing megakaryocytes. No chromosomal abnormalities were detected. Bone marrow immunophenotyping showed no significant abnormalities or immature cell populations suggestive of leukemia. Bone marrow biopsy revealed active proliferation of hematopoietic tissue. Based on her medical history and bone marrow findings, she was diagnosed with ITP. Traditional Chinese medicine was administered at that time, and her platelet count fluctuated between 31 × 10^9^ and 35 × 10^9^/L.

In September 2021, her platelet count decreased to 11 × 10^9^/L. After receiving high-dose dexamethasone pulse therapy and subcutaneous injections of rhTPO (15,000 units/day for 7 days per month), her platelet count significantly increased to the normal range within 1 week. However, when dexamethasone was tapered and rhTPO was discontinued, her platelet count rapidly declined to 14–15 × 10^9^/L.

By mid-October 2021, the patient was started on a standard oral dose of eltrombopag, but her platelet count continued to fluctuate between 7 × 10^9^/L and 14 × 10^9^/L.

From December 2021 onward, she was treated with a combination of eltrombopag, cyclosporine (100 mg every 12 hours), methylprednisolone (12 mg orally twice daily, gradually tapered to discontinuation), and retinoic acid (10 mg twice daily) for 2 months. IVIG therapy (0.4 g/kg, days 1–5) was added during this period when her platelet count dropped below 1 × 10^9^/L. Despite these treatments, her platelet count did not exceed 30 × 10^9^/L.

In March 2022, the patient was treated with low-dose DAC (5.7 mg daily; 3.5 mg/m² per day, intravenous infusion) for 3 consecutive days, administered in 3 cycles (each cycle lasting 21 days). Her platelet count rose to a maximum of 44 × 10^9^/L. On June 23, 2022, she was transferred to our hospital for further treatment. The patient was not taking any medications known to cause thrombocytopenia.

The patient had no significant medical history and denied a history of hypertension, diabetes, liver disease, kidney disease, or other chronic conditions. Physical examination revealed petechiae on the skin of both lower limbs, with no evidence of lymphadenopathy, hepatosplenomegaly, or lower sternal tenderness.

Upon admission, a routine blood test showed a white blood cell count of 6.1 × 10^9^/L, a lymphocyte level of 2.5 × 10^9^/L, a hemoglobin concentration of 135 g/L, and a platelet count of 18 × 10^9^/L. Ferritin, folate, and vitamin B12 levels were within normal ranges. Biochemical testing revealed no significant abnormalities. Antinuclear antibodies and antineutrophil cytoplasmic antibodies were also within normal limits. The patient underwent a repeat bone marrow examination. The bone marrow smear showed a moderate number of nucleated cells and a moderate number of megakaryocytes with poor platelet-producing function. Bone marrow biopsy revealed active hematopoietic tissue proliferation, a slight increase in megakaryocyte count, and no evidence of dysplastic hematopoiesis. Flow cytometry, chromosomal analysis, and gene expression profiling associated with MDS showed no abnormalities.

**Figure 3.**
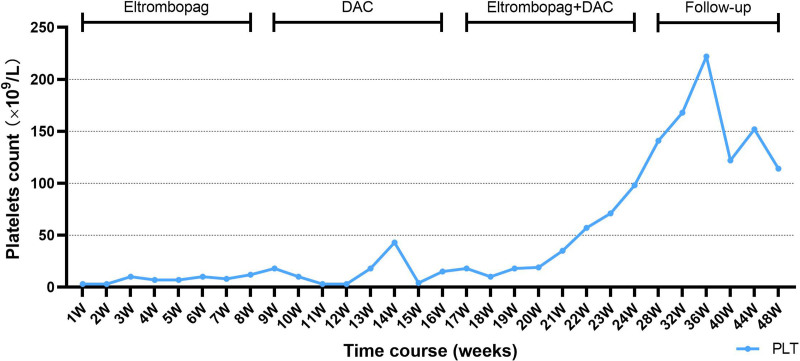
The patient exhibited a poor platelet response during the initial treatment with eltrombopag, as noted during the first week of treatment. Low-dose DAC was initiated at week 9, leading to a rise in platelet count by week 12, followed by a decline at week 14, with persistently low platelet response thereafter. At week 17, combination therapy with eltrombopag and DAC was administered for 8 weeks, during which platelet counts began to rise at week 20 and reached normal levels (>100 × 10^9^/L) by week 24. At 3 months of follow-up, platelet counts remained within the normal range, although minor fluctuations were observed. DAC = low-dose decitabine.

On June 1, 2022, the patient was treated with low-dose DAC (5.7 mg daily; 3.5 mg/m² per day, intravenous infusion) for 3 days combined with eltrombopag (50 mg/day), with a treatment cycle lasting 21 days. After 2 treatment cycles, the patient’s platelet count gradually increased to within the normal range. Although no adverse events related to DAC were observed, the patient discontinued DAC after 2 cycles due to perceived resistance and continued with single-agent eltrombopag maintenance therapy. Her platelet count remained stable within the normal range until May 2023 (Fig. 3).

## 4. Discussion

ITP is an autoimmune-mediated disorder caused by antibody-mediated platelet destruction and impaired platelet production by megakaryocytes.^[1]^ The primary goal of ITP treatment is to elevate platelet levels to prevent bleeding and hemorrhage while minimizing side effects. Refractory ITP is defined as ITP that does not respond to or relapses after splenectomy and requires additional treatments.^[5,6]^ Nonresponsiveness to treatment necessitates a reassessment of the diagnosis to differentiate refractory ITP from secondary ITP and MDS, as well as from hereditary thrombocytopenia, drug-induced thrombocytopenia, and thrombocytopenia caused by bone marrow failure.^[7–9]^ Both patients described in this report failed to benefit from treatment with glucocorticoids, rhTPO, eltrombopag, and IVIG. Refractory ITP remains a significant challenge for clinicians.

If refractory ITP is suspected and multiple single-agent therapies have failed to sustainably increase platelet counts, combination therapies are often the next step. One commonly used regimen is rituximab (RTX) combined with rhTPO. Zhou et al^[10]^ demonstrated that the combination of low-dose RTX and rhTPO significantly increased the complete response (CR) rate and shortened the time to response compared with RTX monotherapy in corticosteroid-resistant or relapsed ITP. However, the study did not demonstrate a long-lasting benefit. Other combination regimens for refractory ITP, such as cyclosporine A combined with eltrombopag, tretinoin combined with danazol, and tretinoin combined with prednisone, have shown limited short-term efficacy. However, due to the small sample sizes and side effects reported in these studies, their clinical application remains limited.^[11–13]^

Low-dose DAC has been included as a third-line treatment in the Chinese guidelines for the diagnosis and management of adult primary ITP (2020 edition).^[6]^ ITP is not solely caused by antibody-mediated platelet clearance; platelet destruction can also be directly mediated by T cells, as reported in recent studies. Low-dose DAC has been shown to act in ITP by restoring immune tolerance through the rebalancing of regulatory T cells.^[14]^ However, the efficacy of low-dose DAC combined with rhTPO or eltrombopag in the treatment of refractory ITP has not been officially reported. DAC, a deoxyribonucleoside phosphorylated by various kinases and incorporated into DNA, is approved by the United States Food and Drug Administration for the treatment of MDS.^[8]^ Given its potential in treating hematologic malignancies, DAC is approved for use in higher-risk patients with MDS and as a first-line treatment for patients with acute myeloid leukemia >65 years who are intolerant to intensive chemotherapy. However, its application in other diseases remains very limited.^[9]^ Therefore, few cases have been reported on the clinical use of DAC in patients with ITP. For the 2 patients diagnosed with refractory ITP described in this report, a combination regimen including low-dose DAC was effective.

A study using may play a role a mouse megakaryoblastic cell line suggested that DNA demethylation in megakaryocyte differentiation and maturation, indicating that treatments targeting DNA methylation could promote platelet release.^[15]^ DAC, as a demethylating agent, has been shown to promote cell differentiation and maturation, improving outcomes in patients with ITP when administered at low doses.^[14,16]^ A prospective, multicenter study on the use of low-dose DAC in adult patients with refractory ITP reported that CR was achieved in 8 patients (17.78%), while partial response was observed in 15 patients (33.33%). The median time to initial response was 28 days (range, 14–70days).^[10]^ Both experimental and clinical studies have demonstrated that low-dose DAC can improve platelet counts. On the other hand, Di Buduo et al^[17]^ highlighted the importance of TPO-RAs in the treatment of ITP, emphasizing their role in both combination and single-agent therapies through the activation of the AKT and ERK1/2 signaling pathways.

Therefore, the 2 patients attempted treatment with low-dose DAC combined with either rhTPO or eltrombopag to improve platelet counts, both achieving a CR. In case 1, following 2 courses of low-dose DAC monotherapy, the patient’s platelet count began to decline. Platelet counts might have returned to the normal range if the patient had continued receiving low-dose DAC combined with rhTPO. This observation suggests a possible synergistic effect of low-dose DAC and rhTPO. In case 2, the patient received low-dose DAC combined with eltrombopag, achieving a normal platelet count after 2 treatment courses. The platelet count remained stable after maintenance therapy with eltrombopag alone, possibly due to the reactivation of the AKT and ERK1/2 signaling pathways. However, whether low-dose DAC combined with other TPO-RAs would yield similar efficacy requires further investigation with additional cases.

## 5. Conclusion

Low-dose DAC combined with rhTPO or eltrombopag may be effective in the treatment of refractory ITP. However, the efficacy of this combination regimen requires further validation in larger studies, and the underlying mechanisms need to be more thoroughly explored. Nonetheless, the success observed in these 2 cases provides a promising, safe, and effective treatment option for patients with refractory ITP.

## Author contributions

**Writing – original draft:** Yuxia Jiang, Yani Xu.

**Writing – review & editing:** Yani Xu.

**Investigation:** Haiying Wu, Minxia He.

**Supervision:** Huifang Jiang, Xiaofeng Xu.

**Project administration:** Xiaofeng Xu.
